# Drosophila Optimization Algorithm Based on Chaotic Development Mechanism and Orthogonal Learning Strategy for Reservoir Optimization

**DOI:** 10.3390/biomimetics11060430

**Published:** 2026-06-17

**Authors:** Rong Lv, Guofa Lei, Hanchao Liu, Yuhan Sun, Wenhua Wang, Xuebin Du

**Affiliations:** 1 Wuhan Geological Survey Center of China Geological Survey (Central South Geological Science and Technology Innovation Center), Wuhan 430205, China; lvrong@mail.cgs.gov.cn; 2 Gas Production Plant 5, Changqing Oilfield Company of PetroChina, Xi’an 710018, China; lgfa1_cq@petrochina.com.cn (G.L.); lhc2_cq@petrochina.com.cn (H.L.); sunyuh_cq@petrochina.com.cn (Y.S.); 3 China Petroleum Group Oriental Geophysical Exploration Co., Ltd., Research Institute Dagang Branch, Tianjin 300280, China; wenhua_wangwork@163.com; 4 College of Oceanography, China University of Geosciences, Wuhan 430074, China

**Keywords:** fruit fly optimization algorithm, production optimization, chaotic exploitation mechanism, reservoir production, industrial production

## Abstract

Enhancing oil and gas production performance is essential for maintaining the economic sustainability of petroleum enterprises and meeting the increasing global energy requirements. In this context, subsurface production optimization constitutes a fundamental component of strategic reservoir management, directly affecting critical decisions such as well location design and the regulation of operational parameters. Nevertheless, conventional reservoir optimization approaches are frequently constrained by high computational costs and limited optimization effectiveness. To overcome these limitations, evolutionary algorithms have gained considerable attention for addressing complex optimization tasks, owing to their gradient-free nature and strong capability for parallel exploration. This paper proposes a chaotic exploitation orthogonal learning fruit fly optimization algorithm (COFOA) tailored for global optimization and oil and gas production optimization. Specifically, we integrate a chaotic exploitation mechanism and an orthogonal learning strategy to improve the balance between exploration and exploitation. Following the population update in FOA, the chaotic exploitation mechanism is first applied to help the population escape local optima and enhance search efficiency. Subsequently, the orthogonal learning strategy is employed to strengthen the algorithm’s exploitation capability. To evaluate the performance of the improved FOA, extensive experiments were conducted on benchmark functions from IEEE CEC 2017 and IEEE CEC 2022, including ablation studies, scalability tests and comparisons with state-of-the-art algorithms. The results demonstrate that the proposed FOA significantly outperforms competing algorithms in optimizing reservoir production. COFOA demonstrates consistent performance superiority over all compared algorithms in terms of mean NPV. Specifically, it achieves improvements of approximately 2.35% to 16.23% compared with existing methods. Notably, COFOA outperforms strong competitors such as mSCA and BLPSO by 2.35% and 3.81%, respectively, while achieving more significant gains over algorithms such as SCADE (15.31%) and CCMSCSA (16.23%). Even when compared with relatively competitive methods like HGWO and CCMWOA, COFOA still maintains performance improvements of 4.79% and 6.12%, respectively. These results clearly demonstrate the superior optimization capability of COFOA in terms of maximizing NPV under complex reservoir conditions.

## 1. Introduction

Reservoir management (RM) has become a major focus within the petroleum industry, drawing increasing attention from oil and gas operators. As defined by Wiggins and Startzman [1], RM refers to the coordinated application of technological capabilities, financial assets, and human resources to enhance both the economic performance and ultimate recovery of a reservoir. They further characterize RM as a holistic and long-term process that covers the entire life cycle of a reservoir, from its discovery stage to final abandonment. Under this perspective, production optimization represents a central element of reservoir management, as petroleum companies aim to improve hydrocarbon recovery not only to satisfy the rising global energy demand but also to maximize economic profitability. Among various production enhancement techniques, waterflooding (also known as water injection) is one of the most extensively utilized methods for improving recovery efficiency. This technique is generally implemented following the primary recovery phase, which primarily depends on natural reservoir drive mechanisms, including gas cap expansion and gravity drainage [2]. Given its significant impact on recovery performance and operational expenses, the design and implementation of waterflooding operations require careful consideration. Consequently, the optimization of waterflooding strategies has remained an active research topic for several decades, providing valuable guidance for petroleum companies in improving recovery efficiency and reducing production costs.

In the context of waterflooding and oil recovery, primary recovery refers to the extraction of hydrocarbons through natural geological and physical forces. The most common recovery mechanisms include gas cap drive, which utilizes the pressure from a gas cap at the reservoir’s crest to push oil toward production wells; gravity drainage, which relies on density differences between oil and water, where injected water displaces oil upward, particularly effective in inclined or vertical reservoirs; and waterflooding, a widely employed enhanced oil recovery (EOR) technique that injects water to maintain pressure and mobilize trapped oil. Additionally, gas injection enhances oil mobility by reducing viscosity, making it particularly effective in high-viscosity reservoirs, while steam injection is primarily used for heavy and highly viscous oils, where steam reduces viscosity and improves flow. Finally, chemical flooding involves the injection of surfactants or polymers to modify interfacial properties, enhancing recovery in high-viscosity reservoirs. Each of these recovery methods offers distinct advantages and limitations, with their selection depending on reservoir characteristics, economic considerations, and technical feasibility.

Waterflooding optimization is categorized as an engineering challenge that requires mathematical algorithms to determine design parameters aimed at maximizing or minimizing predetermined objective functions [3]. These parameters typically include well production rates, well injection rates, bottomhole pressure, and the start time for waterflooding. Additionally, waterflooding can be formulated as a multi-objective optimization problem, where multiple objective functions are optimized simultaneously [4,5,6]. This perspective provides chemical and petroleum engineers with a more comprehensive understanding that aligns closely with real-world operational constraints.

Numerical reservoir simulation (NRS) is one of the most widely used tools in reservoir modeling during the field development stage and can be integrated with various algorithms to address production optimization challenges. However, a major limitation of NRS is the significant computational effort required when modeling geologically complex reservoirs. This is primarily because NRS relies on mathematical equations and physics-based models to simulate subsurface fluid flow, resulting in increased computational time as reservoir complexity escalates. Addressing this computational challenge has become a key focus of research in reservoir engineering.

Conventional gradient-based optimization techniques often face substantial difficulties when applied to global optimization problems that exhibit strong nonlinearity and a large number of local optima. In response to these challenges, a wide range of meta-heuristic optimization methods with diverse search mechanisms have been developed over the past several decades. Owing to their gradient-free characteristics, ease of implementation, and relatively low computational cost, meta-heuristic approaches have been widely adopted for solving complex global optimization problems. Representative examples of classical meta-heuristic algorithms include the moth–flame optimization (MFO) algorithm [7], particle swarm optimization (PSO) [8], bat algorithm (BA) [9], sine–cosine algorithm (SCA) [10]. Among swarm intelligence methods, the fruit fly optimization algorithm (FOA), proposed by Pan in 2012 [11], has attracted attention for mimicking the food-searching behavior of fruit flies through visual and olfactory sensing mechanisms. Despite their success, the No Free Lunch (NFL) theorem formally states that no optimization algorithm can consistently outperform all others across every class of problems [12]. Consequently, a large number of evolutionary and swarm-based algorithms have been reported in the literature [13]. An optimizer that demonstrates strong performance on a particular problem may still be prone to premature convergence or inaccurate solutions when applied to different problem domains. For example, algorithms that perform effectively in continuous optimization tasks may exhibit poor performance in binary feature selection problems. Moreover, swarm-based optimizers often show unstable behavior due to difficulties in maintaining an appropriate balance between exploration and exploitation. Nevertheless, achieving robust optimization performance does not necessarily require the design of a completely new algorithm. In line with the implications of the NFL theorem, enhancing or modifying the search operators of an existing optimizer for a specific class of problems can effectively alleviate its inherent weaknesses and lead to a more stable and competitive variant.

Thus, despite the availability of alternative metaheuristics [14,15], our goal is to enhance the well-established FOA for more stable and efficient performance. FOA is a population-based intelligent optimization algorithm inspired by the foraging behavior of fruit flies. Compared to other optimization algorithms, FOA boasts a simple algorithmic structure, making it easy to implement and debug. By simulating the movement of fruit flies in search of food sources and navigating their environment, FOA effectively performs global searches while minimizing the risk of premature convergence to local optima. The algorithm can adjust its search strategy based on changes in food source concentration, showcasing its adaptability and suitability for dynamic optimization problems. FOA excels at solving multimodal optimization problems with multiple local optima by leveraging collective behavior within the population to explore a broader range of solutions. Its adaptability allows it to quickly adjust search strategies in response to changing conditions in dynamically evolving environments. Furthermore, FOA performs well in high-dimensional search spaces, effectively exploring solutions across multiple dimensions. It is also capable of handling complex constraints, efficiently searching for optimal solutions that satisfy these constraints.

A number of studies have investigated the application of evolutionary and swarm-based optimization techniques to oil and gas production problems. For instance, Wang et al. [16] proposed a robust and efficient framework for real-time production optimization under uncertainty. In their study, the production optimization task is formulated as a Markov decision process, in which a reinforcement learning agent interacts with a reservoir simulator to learn a control policy that maximizes a predefined objective function. To improve the training effectiveness of the intelligent agent, a population-based evolutionary algorithm is incorporated to mitigate premature convergence and insufficient exploration commonly encountered in reinforcement learning approaches. By introducing population diversity and redundant search behaviors, the algorithm enhances the robustness and stability of the learning process. Du et al. [17] developed a model-assisted optimization framework that integrates Bayesian random forest modeling with particle swarm optimization (PSO). Specifically, the Bayesian random forest is utilized to construct a surrogate agent for the injection–production system, enabling accurate prediction of dynamic production parameters based on injection data and operational indicators. On the basis of this surrogate model, PSO is employed to identify optimal injection strategies, while Pareto front analysis is adopted to handle the associated multi-objective optimization problem. Similarly, Ng et al. [18] combined intelligent agents with PSO for production optimization and demonstrated the use of long short-term memory networks to build agents for three-dimensional reservoir modeling. In their approach, a sampling strategy is applied to generate a large number of simulation scenarios, which are subsequently used as training data for agent development.

Existing studies on the FOA and its variants can be broadly categorized into three main research directions, reflecting the evolution of improvement strategies. The first category focuses on chaotic strategy-based enhancements, where chaotic maps replace random initialization or perturbation processes to improve population diversity and alleviate premature convergence. The second category involves orthogonal learning-based approaches, which employ orthogonal experimental design to systematically sample the search space, thereby improving solution quality and convergence accuracy. The third category consists of hybrid and composite strategies, in which FOA is combined with other optimization mechanisms to enhance convergence speed and robustness.

From a developmental perspective, early studies mainly emphasized enhancing global exploration through randomness and chaos, whereas more recent works have shifted toward structured learning mechanisms and hybrid frameworks to strengthen exploitation and overall search efficiency. However, most existing methods rely on a single improvement strategy, which limits their ability to simultaneously achieve strong exploration and effective exploitation. This limitation becomes particularly critical in complex real-world optimization problems.

Motivated by these observations, this paper proposes a chaotic exploitation orthogonal learning fruit fly optimization algorithm (COFOA), which integrates a chaotic exploitation mechanism with an orthogonal learning strategy within a unified framework. Unlike existing approaches that treat these strategies independently, the proposed method tightly couples them to enhance both search diversity and solution refinement. This design enables more efficient use of limited evaluations and improves the balance between global exploration and local exploitation.

Furthermore, according to the No Free Lunch (NFL) theorem, no optimizer performs best across all problem domains. However, its performance can be significantly improved for specific classes of problems through tailored enhancements. In this context, instead of developing an entirely new meta-heuristic, this study focuses on systematically improving the well-established FOA to better suit expensive simulation-based optimization tasks. The proposed method aims to provide a more stable, efficient, and practically applicable optimization framework for real-world engineering problems, particularly in oil and gas production optimization.

The proposed COFOA in this study achieved the best results, ranking first among other advanced competitors in both IEEE CEC 2017 and IEEE CEC 2022. In IEEE CEC 2017, COFOA demonstrated a significant advantage over the second-ranked CLACO across 17 test functions. Similarly, in IEEE CEC 2022, COFOA outperformed the second-ranked QCSCA in six test functions, showcasing its superior performance.

- This study introduces an enhanced version of the Fruit Fly Optimization Algorithm (FOA), referred to as COFOA, which incorporates chaotic exploitation and an orthogonal learning strategy.
- To evaluate the performance of the proposed COFOA, it is systematically compared with several state-of-the-art evolutionary algorithms using benchmark test suites from the IEEE CEC 2017 and IEEE CEC 2022 competitions. In addition, an in-depth investigation is conducted to examine the individual contributions of the two enhancement mechanisms to the overall performance of COFOA, as well as its scalability under different problem dimensionalities.
- To validate the applicability of the proposed COFOA to practical production optimization tasks, it is employed to solve a real-world production optimization problem in three-channel reservoir systems. Furthermore, its performance is benchmarked against several state-of-the-art evolutionary algorithms. The experimental findings confirm the superior optimization effectiveness of COFOA in realistic engineering scenarios.

This study is organized as follows. Section 2 provides an in-depth overview of the FOA. Section 3 details the integration of the two new mechanisms into the FOA. In Section 4, a series of comparative experiments are performed on the COFOA. Finally, Section 5 concludes the chapter and highlights directions for future research.

## 2. Fruit Fly Optimization Algorithm

### 2.1. Fruit Fly Optimization Algorithm

The Fruit Fly Optimization Algorithm (FOA), proposed by Pan [11], is a bio-inspired optimization technique derived from the collective foraging behavior of fruit fly swarms. In FOA, candidate solutions are explored within the search space by emulating the dual sensory mechanisms of fruit flies, namely olfaction and vision. At the early stage of the search, fruit flies are randomly dispersed in various directions and step sizes, relying primarily on their olfactory sense to detect potential food sources. As the search progresses and promising regions are identified, visual sensing is activated to enable more accurate localization.

During this process, each fruit fly evaluates the quality of the sampled position, and information sharing within the swarm allows the identification of the location associated with the highest food concentration. The swarm then collectively moves toward this best position, guiding the subsequent search. Once the current optimal region is reached, the population continues to explore its neighborhood in order to discover areas with even higher food concentrations, thereby iteratively refining the solution.

In FOA, the population update process is a core mechanism that simulates the behavior of fruit flies in their search for food sources. Initially, the algorithm generates a specified number of fruit flies at random positions, with each fly’s initial position representing a solution in the search space. During each iteration, the fruit flies evaluate the food source concentration at their current positions, typically determined by the objective function value. Based on the best food source identified in the current iteration, the fruit flies move towards that position, thus updating their locations. To enhance search diversity, the algorithm incorporates a random walk mechanism, allowing fruit flies to maintain some degree of randomness in their movements to avoid premature convergence. By iteratively repeating this process, FOA effectively explores and converges towards the global optimum in the search space, ultimately providing the position of the best fruit fly along with its corresponding concentration value as the optimal solution to the objective function. This strategy, which combines focused search with random exploration, proves effective for FOA when addressing complex optimization problems.

The FOA procedure begins with a parameter initialization stage. Specifically, the maximum number of fitness evaluations (MaxFEs), population size (popsize), and the allowable search range for population positions (LR) are first specified. Subsequently, the initial locations of all fruit fly individuals are randomly generated within the predefined search space.

(1) $$X_{axis}=rand(LR)$$

(2) $$Y_{axis}=rand(LR)$$

Initialize population. The fruit fly individuals engage in random olfactory-based food searches. In the FOA, each individual in the population represents a candidate solution in the search space. The coordinates $X_{axis}$ and $Y_{axis}$ denote the spatial positions of an individual in a two-dimensional solution space, corresponding to the values of the decision variables for that solution. Essentially, each fruit fly’s location encodes a potential solution that can be evaluated by the objective function. The population in FOA consists of multiple such individuals, each with its own coordinates, allowing the algorithm to explore and exploit the search space collectively. Thus, $X_{\mathrm{axis}}$ and $Y_{\mathrm{axis}}$ are fundamental to representing and updating the positions of the population members during the optimization process. In the FOA, the representation naturally extends from two-dimensional space to high-dimensional problems without altering the core mechanism. For a D-dimensional optimization task, each individual in the population is modeled as a D-dimensional position vector $X_{i}=[x_{i1},x_{i2},\ldots ,x_{iD}]$, where each component corresponds to a decision variable in the problem. The population therefore consists of multiple such vectors, forming a set of candidate solutions distributed in the D-dimensional search space. During the optimization process, the position of each individual is updated independently along each dimension through random perturbations or search strategies, enabling exploration and exploitation of the solution space. The fitness of each individual is then evaluated based on its position vector, and the population collectively evolves toward better regions. Thus, the traditional $X_{\mathrm{axis}}$ and $Y_{\mathrm{axis}}$ in FOA can be generalized to a multi-dimensional coordinate system, where each dimension represents a variable, and the population corresponds to a set of such high-dimensional solution vectors.

(3) $$X_{i}=X_{axis}+tempx$$

(4) $$Y_{i}=Y_{axis}+tempy$$

(5) $$tempx=rand\;in\;\left[-10,10\right]$$

(6) $$tempy=rand\;in\;\left[-10,10\right]$$

Population assessment. Initially, the distance from each fruit fly individual in the population to the initial position can be evaluated using the following formula.

(7) $${Dist}_{i}=\sqrt{X_{i}^{2}+Y_{i}^{2}}$$

Next, utilize the reciprocal of the distance as the measure for determining odor concentration.

(8) $$S_{i}=1/{Dist}_{i}$$

Calculate the fitness value. Replace the odor concentration $S_{i}$ within the odor concentration determination function to determine the odor concentration ${Smell}_{i}$ at the fruit fly’s location.

(9) $${Smell}_{i}=Fitness\;function(S_{i})$$

Find out optimal. Seek fruit flies exhibiting optimal odor concentration levels.

(10) $$\left[bestSmell\;bestindex\right]=min(Smell)$$

Retain concentration values and coordinates. While the fruit fly population employs vision to navigate to the location, the optimal odor concentration value and the fruit fly’s location information remain unchanged.

(11) $$X_{axis}=X(bestindex)$$

(12) $$Y_{axis}=Y(bestindex)$$

(13) Smellbest=bestSmell

Iteration process. Repeat steps 2–5 if the current odor concentration value is lower than the odor concentration value of the previous generation. Otherwise, proceed to step 6. The implementation of FOA can be described as Algorithm 1. For time complexity, the time complexity of FOA is *O*(FOA) = *O*(initialization) + *O*(Population update) ≈ *O*(*N* × *D*) + *O*(*N* × *D* × *T*) ≈ *O*(*N* × *D* × *T*).
**Algorithm 1** Pseudo-code of FOA1. Set the initial values for the parameters *popsize* and *MaxFEs*.2. Establish the population of fruit flies with their respective *X_axis* and *Y_axis* positions.3. For i=1 to popsize4.   Compute the initial fitness scores.5. End For6. $\left[bestSmell\;bestindex\right]=\min\left(Smell\right);$7. $X_{axis}=X\left(bestindex\right);$8. $Y_{axis}=Y\left(bestindex\right);$9. bestCVaccuarcy=bestSmell;10.While (iteration≤Max_iteraition)11.  For i=1 to popsize12.       $X_{i}=X_{axis}+randomValue;$13.       $Y_{i}=Y_{axis}+randomValue;$14.       ${Dist}_{i}=\sqrt{X_{i}^{2}+Y_{i}^{2}};$15.       $S_{i}=1/{Dist}_{i};$16.       $Smell\left(i\right)=Function\left(S_{i,j}\right);$17.       $\left[bestSmell\;bestindex\right]=\min\left(Smell\right);$18.       If bestSmell<bestCVaccuarcy19.         $X_{axis}=X\left(bestindex\right);$20.         $Y_{axis}=Y\left(bestindex\right);$21.         bestCVaccuarcy=bestSmell;22.       End If23.  End For24.iteration=iteration+1;25.End While26.Return bestCVaccuarcy;

### 2.2. Related Work

In this section, we will introduce research related to swarm intelligence algorithms.

Zhou et al. [19] introduced an improved version of the Tree Seed Algorithm (TSA) that combines the water cycle and quantum rotation gate mechanisms. These enhancements help the algorithm overcome local optima and strike a better balance between exploration and exploitation. By incorporating physical mechanisms such as water cycling and quantum rotation gates, Zhou et al. enhanced the global search capabilities and local optimum handling of TSA, emphasizing the simulation of physical phenomena to achieve an efficient optimization process. Chen et al. [20] introduced two new mechanisms to improve the exploration and development strategies of the original Fruit Fly Optimization Algorithm (FOA), the sentinel mechanism and the multi-population mechanism. The sentinel mechanism consists of two parts, greedy selection and Gaussian mutation, which primarily enhance the convergence speed of the algorithm. The multi-population mechanism divides the population of intelligent agents into multiple subgroups, from which individuals are randomly selected. This strategy increases the diversity and exploration capacity of the algorithm. By introducing the forerunner mechanism and multi-population strategy, Chen et al. aimed to enhance search efficiency and adaptability. The forerunner mechanism helps fruit flies move more rapidly toward high-quality solutions, while the multi-population strategy boosts the algorithm’s diversity and exploratory capabilities.

The proposed Chaotic Exploitation Orthogonal Learning Fruit Fly Optimization Algorithm (COFOA) incorporates chaotic mechanisms that allow the algorithm to dynamically adjust its search paths, enhancing its global search capabilities. The orthogonal learning mechanism aids fruit flies in learning more effectively by reducing interference between features, improving the overall efficiency of the algorithm. This combination of mechanisms enables FOA to exhibit greater flexibility and adaptability in complex and dynamic environments. By optimizing feature independence, the orthogonal learning strategy improves the algorithm’s learning efficiency, allowing it to find optimal solutions more rapidly in complex feature spaces. Furthermore, COFOA has been applied to real oil reservoir production, demonstrating the algorithm’s practical applicability.

Abed-alguni et al. [21] introduced a CS variant called Exploratory CS (ECS), which made three modifications to the original CS algorithm to enhance its exploratory ability. Firstly, ECS uses a special type of adversarial learning called refraction learning to enhance CS’s ability to escape suboptimal states. Secondly, ECS uses Gaussian perturbation to optimize the worst candidate solution in the population before the dropout step in CS. Thirdly, in addition to the Lévy flight mutation method used in CS, ECS also employs two mutation methods, namely highly destructive polynomial mutation and Jaya mutation, to generate new improved candidate solutions.

## 3. The Proposed COFOA

The original FOA is advantageous due to its simple structure, minimal control parameters, and ease of understanding. However, its convergence speed and accuracy on multi-modal and complex functions are suboptimal. To address these limitations, this paper introduces two enhanced mechanisms designed to improve both the convergence speed and solution quality of the classical FOA.

### 3.1. Chaotic Exploitation

The state of chaos in nonlinear systems is characterized by irregularity, combining disorder with internal order and blending certainty with randomness in system behavior. Mathematically, chaotic systems can be viewed as sources of randomness. Chaotic motion within a defined range can traverse all states of the space according to its own laws. In contrast to probabilistic random search strategies, exploiting chaos reduces randomness and enhances search efficiency. Furthermore, chaotic sequences are easy to generate and store, allowing for the easy acquisition of diverse sequences through adjustments in initial conditions. These sequences are deterministic and repeatable, generated by Equation (14) using logistic mapping. Chaotic mechanism plays a critical role in enabling the population to escape from local optima by enhancing the diversity and ergodicity of the search process. Chaotic systems exhibit deterministic yet pseudo-random behavior, characterized by irregularity that combines disorder with inherent structure. This property allows chaotic sequences to traverse the search space more uniformly compared to purely stochastic random strategies. Unlike conventional random perturbations, chaotic perturbation reduces redundant exploration and improves search efficiency by guiding individuals toward unexplored regions. In addition, chaotic sequences are highly sensitive to initial conditions, making them easy to generate while providing diverse and repeatable search trajectories.

(14) $$C_{i+1}=\gamma \times C_{i}\times \left(1-C_{i}\right)\;\;\;i=1,\cdots ,n-1$$

Set γ=4, and let $C_{1}\in \left(0,1\right)\cup C_{1}\neq 0.25\cup C_{1}\neq 0.5\cup C_{1}\neq 0.75\cup \;C_{1}\neq 0\;\cup \;C_{1}\neq 1$. When γ=4, the logistic mapping exhibits complete chaos. Here, *n* denotes the number of fruit fly. $C_{i}$ represents an individual within the population in optimization algorithms.

Optimizing chaotic exploitation within a limited range is feasible. However, as the search space expands excessively, the associated time costs become prohibitive. Therefore, integrating chaotic exploitation into other heuristic algorithms presents a viable approach. Candidate solutions for the target position generated by the Chaotic Learning Strategy (CLS) are:

(15) $$CS=\left(1-s\right)\times T+s\times C_{i}^{\prime }\;\;\;\;i=1,\cdots ,n$$

The constriction factor s is defined as follows:

(16) s=(G−g+1)/G

(17) $$C_{i}^{\prime }=lb+C_{i}\times \left(ub-lb\right)$$

The chaotic variable $C_{i}$ in Equation (14) comprises Equation (11), and the chaotic vector $C_{i}^{\prime }$ is constrained within the range [lb, ub], where lb, and ub denote the boundaries of the fruit fly. lb represents the minimum value, and ub the maximum. The candidate solution CS is obtained through a linear combination of the chaotic vector $C_{i}^{\prime }$ and the target position T.

In this study, the Logistic map is employed as a chaotic mapping technique. The Logistic map is a simple yet powerful tool, widely used in evolutionary algorithms due to its effectiveness. It generates values through an iterative process, exhibiting chaotic behavior at specific parameter values. The output of the Logistic map is highly sensitive, where even slight variations in initial conditions can lead to drastically different outcomes. This characteristic results in a highly irregular sequence, which in turn increases search diversity.

The integration of the Logistic map into evolutionary algorithms can significantly enhance global search capabilities. By generating random sequences that span the entire search space, the algorithm is less likely to become trapped in local optima during the search process. The chaotic nature of the map adds a dynamic aspect to the search, enabling the algorithm to adapt its direction flexibly and find better solutions. Furthermore, the Logistic map improves the convergence speed of the algorithm. When combined with other optimization strategies, it helps mitigate the risk of converging to suboptimal solutions. As such, the Logistic map serves as an important tool for improving the performance of evolutionary algorithms, particularly in tackling complex and multimodal optimization problems, thereby enhancing both the effectiveness and efficiency of the algorithm. In the proposed COFOA, the chaotic sequence is applied to all individuals in the population during the chaotic exploitation phase. Specifically, after each iteration, the position of every fruit fly is updated by incorporating a chaotic perturbation based on its current coordinates, rather than applying chaos only to the current best or a random subset of individuals. This design ensures that the diversity of the population is preserved and that the algorithm can explore a broader region of the search space while simultaneously refining candidate solutions around promising areas. By uniformly introducing chaotic dynamics across all individuals, COFOA enhances the global exploration capability and reduces the likelihood of premature convergence, thereby improving both robustness and convergence quality. In COFOA, the chaotic parameters used in the chaotic exploitation mechanism are initialized using uniform random sampling within predefined bounds that correspond to the feasible search space of each decision variable. This ensures that the initial chaotic sequence starts from valid positions and fully covers the solution domain. During iterations, chaotic values are generated using a deterministic chaotic map, and each new candidate position is checked against the variable bounds. If a candidate value falls outside the permissible range, it is either clipped to the nearest boundary or reinitialized randomly within the feasible range. This approach guarantees that all generated positions remain valid throughout the optimization process, preventing illegal solutions from corrupting the search and maintaining the integrity of both global exploration and local exploitation. By systematically initializing chaotic parameters and controlling forbidden values, the algorithm achieves stable and robust performance while fully leveraging the advantages of chaotic dynamics.

### 3.2. Orthogonal Learning

The orthogonal learning strategy mimics orthogonal experimental design to identify the optimal combination efficiently. OED aims to achieve optimal experimental outcomes with minimal tests, based on predefined factors and their respective levels. Consider an experiment with Q levels and F factors. To determine the best combination among all $Q^{F}$ possible combinations, conducting experiments for each orthogonal combination would require $Q^{F}$ trials. However, orthogonal arrays provide a method to identify the optimal combination using a reduced and representative experimental design. $L_{M}\left(Q^{F}\right)$ denotes the orthogonal array (OA) for this factor and scenario.

An orthogonal array with F factors and Q levels per factor can be denoted by $L_{M}\left(Q^{F}\right)$, where M represents the minimum number of test combinations required. For example, $L_{9}\left(3^{4}\right)$ is illustrated below:

(18) $$\left[\begin{array}{cccc} 1\; & 1 & 1 & 1\\ 1 & 2 & 2 & 2\\ 1 & 3 & 3 & 3\\ 2 & 1 & 2 & 3\\ 2 & 2 & 3 & 1\\ 2 & 3 & 1 & 2\\ 3 & 1 & 3 & 2\\ 3 & 2 & 1 & 3\\ 3 & 3 & 2 & 1 \end{array}\right]$$

The orthogonal array possesses two primary attributes. Firstly, within each column, numbers 1, 2, and 3 appear with equal frequency, ensuring uniform distribution. This characteristic allows each factor level to contribute equally across tests, minimizing cross-factor interference and facilitating effective comparisons. Secondly, every pair of horizontally adjacent columns exhibits equal occurrences of number pairs. This characteristic ensures even distribution of test points across the entire factorial space, enhancing representativeness.

When conducting a test involving all four factors and three levels, traditionally, 3^4^ = 81 experiments would be required. However, employing $L_{9}\left(3^{4}\right)$ necessitates only nine experimental combinations to yield crucial information. Therefore, the orthogonal array significantly reduces experimental resource consumption.

The Orthogonal Learning (OL) mechanism is an efficient search strategy inspired by the principles of Orthogonal Design, aiming to fully exploit useful information from multiple candidate solutions with minimal computational cost to construct better search directions. In traditional evolutionary optimization, individual updates often rely on a single reference solution, such as the current best, which can lead to underutilization of information and premature convergence. In contrast, Orthogonal Learning introduces multiple reference individuals, such as the current individual, the global best, and historically superior solutions. This generates a set of representative candidate solutions that provide good coverage and diversity across the search space. The fitness of these candidates is then evaluated, and the best-performing combination is selected as the new learning direction or to update the individual’s position, enabling a more refined and efficient exploration of the solution space. The advantages of this mechanism include enhanced information recombination, improved exploitation capabilities, and reduced risk of being trapped in local optima while maintaining low computational complexity. By integrating the Orthogonal Learning mechanism, the algorithm achieves higher convergence accuracy, better solution quality, and more stable global optimization performance. In experiments, the key parameters of the orthogonal learning mechanism are typically set as follows: the number of levels Q = 2, to ensure effective coverage of the search space whilst maintaining low computational complexity; the number of factors F is determined adaptively based on the problem dimension D. For low-dimensional problems, F is usually set to D, whereas for high-dimensional problems, a grouping strategy is adopted to reduce computational overhead whilst maintaining good search performance. This parameter configuration has been shown in most swarm intelligence optimization algorithms to strike a good balance between exploration capability and computational efficiency. In the orthogonal learning mechanism of COFOA, orthogonal factors correspond to the decision variables or grouped dimensions of the solution vector that are systematically combined to generate candidate solutions. Each factor represents a particular dimension of the search space, and the term “orthogonal” indicates that the values of different factors are varied independently according to an orthogonal array. By designing candidate solutions such that all possible levels of each factor are evenly and systematically sampled, the algorithm ensures a balanced coverage of the multidimensional search space without requiring an exhaustive combinatorial search. This structured recombination allows COFOA to efficiently explore promising regions by leveraging information from multiple reference solutions, improving convergence speed and solution quality. In practice, the number of orthogonal factors (denoted as F) can be set to the total number of decision variables or to a subset through a grouping strategy, depending on the problem’s dimensionality and computational budget. In the orthogonal learning mechanism of COFOA, orthogonal levels refer to the discrete values or settings that each orthogonal factor can take when constructing candidate solutions. Each factor (representing a dimension or group of dimensions of the solution vector) is assigned multiple levels, typically corresponding to low, medium, and high values, or other representative samples within the feasible range. By combining the levels of all factors according to a predefined orthogonal array, the algorithm generates a set of candidate solutions that systematically and evenly cover the search space. This ensures that all levels of each factor are considered in different combinations, maximizing the information gained from limited evaluations. Proper selection of levels allows COFOA to efficiently explore promising regions while maintaining computational efficiency, effectively enhancing both convergence precision and robustness.

### 3.3. The Proposed COFOA

In this section, the two mechanisms are integrated into the traditional Fruit Fly Optimization Algorithm (FOA), and the entire COFOA procedure is detailed. Within COFOA, a chaotic exploitation mechanism is first employed to enhance the capability of searching for global optimal solutions, while simultaneously applying the orthogonal learning mechanism to accelerate the algorithm’s convergence rate. The chaotic mechanism utilizes the inherent properties of chaotic systems to increase the diversity and unpredictability of the search process. By incorporating chaotic mappings, the algorithm is able to explore the search space more effectively, thereby avoiding premature convergence to local optima. Compared to traditional random search methods, the chaotic mechanism provides a more uniform distribution of samples, thereby improving global optimization capabilities. The orthogonal learning strategy focuses on leveraging orthogonality during the learning process to minimize interference among models. By introducing orthogonal structures, the models can independently learn distinct features, thereby improving both learning efficiency and accuracy. This approach is particularly effective in multi-task learning and feature selection, enhancing the models’ generalization abilities. Conventional evolutionary algorithms, such as genetic algorithms, primarily rely on operations like selection, crossover, and mutation. In contrast, the chaotic mechanism optimizes the search process through dynamically changing strategies. Meanwhile, the orthogonal learning strategy boosts model performance by ensuring feature independence, whereas traditional evolutionary algorithms optimize individuals through fitness evaluations. This gives the orthogonal strategy a distinct advantage in feature selection and model training.

The pseudo-code of COFOA is as presented in Algorithm 2. The COFOA is a bio-inspired metaheuristic method designed for solving optimization problems. Initially, parameters such as population size (popsize) and maximum function evaluations (*MaxFEs*) are set. A population of fruit flies is established, each characterized by $X_{axis}$ and $Y_{axis}$ positions. Fitness scores for each fly are computed iteratively. The algorithm identifies the best-performing fly based on its fitness score. Iterations continue until the maximum number of iterations (Max_iteraition) is reached. During each iteration, each fly’s position is updated by adding random values to their current positions, followed by computing their distance from the origin and subsequent fitness evaluation using a specified function. If a fly’s fitness improves over the current best (bestCVaccuarcy), the position of the best fly and its fitness are updated accordingly. Strategies such as chaotic exploitation and orthogonal learning are employed to update parameters influencing fly behavior, enhancing exploration and exploitation capabilities. The algorithm iteratively refines its search, aiming to converge towards the best fitness solution found. FOA’s methodology offers an effective approach for addressing complex optimization challenges across various domains. After candidate solutions are generated in COFOA, either through random perturbation, chaotic exploitation, or orthogonal learning mechanisms, each candidate is evaluated using the fitness function. The algorithm then identifies the best-performing candidate within the current population by comparing all fitness values. If the best candidate achieves a better fitness than the current global best solution, the global best is updated accordingly, and the corresponding position becomes the new reference for the next iteration. Candidates that do not outperform the global best are retained in the population for further updates in subsequent iterations, maintaining diversity and preventing loss of potentially useful search directions. This approach ensures that COFOA continuously exploits promising regions while preserving exploratory potential, effectively balancing exploitation and exploration throughout the optimization process. For time complexity, the time complexity of COFOA is *O*(COFOA) = *O*(initialization) + *O*(Population update) + *O*(Chaotic exploitation) + *O*(OL) ≈ *O*(*N* × *D*) + *O*(*N* × *D* × *T*) + *O*(*N* × *D*) + *O*(*N* × *D* (*M* + *Q* × *M* + 1)) + *O*(*N* × (*M* + 1)) ≈ *O*(*N* × *D* × *T*) + *O*(*N* × *D* (*M* + *Q* × *M* + 1)) + *O*(*N* × (*M* + 1)).

The exploration and exploitation capabilities of COFOA are explicitly balanced through its iterative search process. In each iteration, the positions of fruit flies are updated by adding random perturbations to the current global best ($X_{\mathrm{axis}},Y_{\mathrm{axis}}$), which allows the algorithm to explore new regions of the search space and prevents premature convergence to local optima, representing the exploration component. At the same time, the algorithm enhances exploitation through two complementary mechanisms: the chaotic exploitation mechanism and the orthogonal learning mechanism. The chaotic exploitation mechanism introduces deterministic but non-repetitive perturbations to the solution, allowing finer local search around promising areas, while the orthogonal learning mechanism systematically combines dimensional information from multiple reference solutions to construct more informative candidate positions. By evaluating the fitness of these candidates and updating the best solution accordingly, COFOA effectively refines high-quality solutions in promising regions. Overall, the random updates ensure sufficient global search (exploration), whereas the chaotic and orthogonal learning updates intensify local refinement (exploitation), enabling COFOA to maintain a proper balance between diversification and intensification and thus achieve high convergence accuracy and robust performance across complex optimization landscapes.

The pseudo-code of the proposed COFOA is summarized as follows. First, the algorithm initializes the key parameters, including the population size (*popsize*) and the maximum number of iterations (*Max_iteration*). A population of fruit flies is then generated by assigning each individual random positions in the two-dimensional search space, denoted by *X_axis* and *Y_axis*. The initial fitness (smell concentration) of each individual is evaluated, and the best individual is identified according to the minimum fitness value. Its corresponding position is recorded as the current global best, and its fitness is stored as *bestCVaccuracy*. Subsequently, the algorithm enters an iterative optimization process. At each iteration, each individual updates its position by performing a random search around the current best location. The Euclidean distance to the origin is computed, and the smell concentration is inversely proportional to this distance, which is then evaluated by the objective function. The best individual within the current population is selected, and if its fitness improves upon the current global best, the global best position and fitness are updated accordingly. After the population update, two enhancement strategies are applied to further refine the search process, namely the chaotic exploitation mechanism and the orthogonal learning mechanism, both of which update the candidate solutions to improve exploration and exploitation capabilities. The iteration counter is then increased, and the procedure repeats until the termination criterion (*Max_iteration*) is satisfied. Finally, the algorithm outputs the best fitness value obtained during the search process.
**Algorithm 2** Pseudo-code of COFOA1. Set the initial values for the parameters popsize and Max_iteraition.2. Establish the population of fruit flies with their respective $X_{axis}$ and $Y_{axis}$ positions.3. For i=1 to popsize4.   Compute the initial fitness scores.5. End For6. $\left[bestSmell\;bestindex\right]=\min\left(Smell\right);$7. $X_{axis}=X\left(bestindex\right);$8. $Y_{axis}=Y\left(bestindex\right);$9. bestCVaccuarcy=bestSmell;10.While (iteration≤Max_iteraition)11.  For i=1 to popsize12.       $X_{i}=X_{axis}+randomValue;$13.       $Y_{i}=Y_{axis}+randomValue;$14.       ${Dist}_{i}=\sqrt{X_{i}^{2}+Y_{i}^{2}};$15.       $S_{i}=1/{Dist}_{i};$16.       $Smell\left(i\right)=Function\left(S_{i,j}\right);$17.       $\left[bestSmell\;bestindex\right]=\min\left(Smell\right);$18.       If bestSmell<bestCVaccuarcy19.         $X_{axis}=X\left(bestindex\right);$20.         $Y_{axis}=Y\left(bestindex\right);$21.         bestCVaccuarcy=bestSmell;22.       End If23.  End For24.  Updating $S_{i,j}$ by chaotic exploitation mechanism;25.  Updating $S_{i,j}$ by orthogonal learning mechanism;26.       iteration=iteration+1;27.End While28.Return bestCVaccuarcy;

## 4. Experimental Results and Analysis

In this section, the effectiveness of the proposed algorithm is thoroughly validated through extensive experimentation, including ablation studies, extension trials, historical search trace analysis, comparative tests with other algorithms, and the application of the proposed COFOA to real-world engineering problems. In the experiments, the following parameter settings were used. The population size was set to 30, and the maximum evaluation count was uniformly set to 300,000. All algorithms were tested 30 times on the benchmark functions. For the orthogonal learning mechanism, *Q* was set to 4 and *F* was set to 5. In Chaotic exploitation, γ=4. The parameter settings are based on experience. All experiments were conducted on a Windows 10 and 16 GB of memory, CPU is Intel(R) Core(TM) i5-8265U CPU @ 1.60GHz. All algorithms were implemented in MATLAB R2014b.

### 4.1. Benchmark Functions

#### 4.1.1. IEEE CEC 2017 Benchmark Functions

Table 1 shows the details of the IEEE CEC 2017 benchmark functions.

#### 4.1.2. IEEE CEC 2022 Benchmark Functions

Table 2 shows the details of the IEEE CEC 2022 benchmark functions.

### 4.2. Ablation Analysis

This section highlights the significant effects of two enhancement mechanisms on COFOA through ablation experiments, which are critical in scientific research. Ablation experiments are essential for validating the robustness and reliability of research outcomes. By systematically removing a variable and examining its impact, these experiments confirm the observed phenomena and rule out other potential explanations. This approach allows researchers to assess the role and significance of each factor in the study, thereby strengthening the credibility of the findings and minimizing confounding variables. Ablation experiments are crucial for testing scientific hypotheses, supporting research conclusions, and enhancing the reliability and reproducibility of results.

Table 3 presents the results, where CFOA represents the FOA improved only by the chaotic exploitation mechanism, and OFOA denotes FOA enhanced solely by the orthogonal learning mechanism. Based on 30 independent experiments on the CEC 2017 benchmark functions, the data show that COFOA, enhanced by both mechanisms, clearly outperforms FOA enhanced by either mechanism alone. Specifically, COFOA excels over CFOA in 8 functions and OFOA in 18 functions, demonstrating that combining both mechanisms significantly improves FOA.

### 4.3. Scalability Analysis

This section examines the scalability of COFOA by varying the problem dimensions during testing. Scalability tests are crucial for evaluating the performance of evolutionary algorithms in handling large-scale problems. By adjusting the size of the problem across different dimensions, the algorithm’s ability to manage varying sizes and complexities can be assessed. These tests evaluate performance based on resource utilization, time consumption, and solution quality, providing insights into the algorithm’s applicability and limitations. Scalability tests are essential for offering reliable solutions to large-scale issues in practical scenarios, thereby supporting the broader adoption of evolutionary computing in real-world applications.

In this study, three dimensions are tested 30, 50, and 100, which are standard benchmarks in evolutionary computing. Table 4 presents the scalability analysis results, where COFOA consistently outperforms FOA across all tested dimensions. The scalability tests include a comparison with the original FOA, demonstrating COFOA’s superior performance in optimizing problems of varying dimensions.

### 4.4. Parameter Sensitivity Analysis

To gain deeper insights into the algorithm’s parameter sensitivity, we examine the effects of population size by varying one parameter at a time while keeping the others constant. In this experimental setup, functions f1, f2, and f3 are selected for evaluation under fixed conditions. The population size is adjusted to 10, 30, 60, 100, and 200 to investigate its influence on algorithmic performance. As shown in Table 5, the proposed COFOA consistently demonstrates superior performance across different population sizes, suggesting its robustness and reduced sensitivity to this parameter. In contrast, FOA exhibit greater fluctuations in performance, indicating a stronger dependence on population size, whereas COFOA maintains greater stability under these variations.

### 4.5. Comparison of Other Related Algorithms

#### 4.5.1. Comparative Experiments at CEC 2017 Benchmark Functions

This section evaluates COFOA using the IEEE CEC 2017 benchmark functions. The Wilcoxon signed-rank test [22] and Friedman test [23] was employed to evaluate performance. To ensure fair comparison, all algorithms were tested under consistent conditions. The population size (popsize) was set to 30, the number of sub-populations was fixed at 3, and the maximum evaluation number (MaxFEs) was uniformly set to 300,000. To ensure a fair and comprehensive evaluation, a diverse set of representative algorithms is selected for comparison. Specifically, the compared methods include classical metaheuristic algorithms, well-established improved variants, and recently proposed state-of-the-art approaches. Classical algorithms are included due to their widespread use and strong baseline performance in optimization tasks. In addition, several enhanced algorithms with advanced search strategies are incorporated to reflect the current development trends in the field. Furthermore, recent high-performance algorithms reported in the literature are also considered to provide a rigorous benchmark against state-of-the-art methods. These algorithms have been widely adopted and validated on standard benchmark problems, which ensures that the comparison is both fair and representative. Therefore, the selected comparison set covers different categories of optimization methods and adequately demonstrates the effectiveness and competitiveness of the proposed COFOA.

Table 6 presents a comprehensive evaluation of the COFOA, benchmarking it against a suite of state-of-the-art algorithms on the IEEE CEC 2017 functions. The competing algorithms involved in this experiment include HGWO [24], WEMFO [25], mSCA [26], SCADE [27], CCMWOA [28], QCSCA [29], BWOA [30], CCMSCSA [31], SSNMRA [32], BLPSO [33], GCHHO [34]. This comparative study aims to assess the efficacy of COFOA in terms of optimization performance, providing insights into its robustness and effectiveness relative to other advanced methods. The COFOA demonstrates a superior performance, achieving the top rank with a significantly lower average score of 1.84 × 10^0^. This indicates its exceptional optimization capability. As the reference algorithm, COFOA’s comparative results are denoted by “~,” reflecting its baseline status in this context. Notably, COFOA’s dominance is evident as it consistently outperforms other algorithms across the evaluated benchmarks, showcasing its robustness and efficiency in solving complex optimization problems. In contrast, the HGWO, ranked 9th, exhibits a markedly lower performance with an average score of 9.12 × 10^0^. The “+/=/−” metric of 30/0/0 indicates that COFOA outperforms HGWO in all benchmark comparisons, underscoring HGWO’s significant shortcomings in this experimental setup. Similarly, SCADE and CCMWOA, which are ranked 11th and 12th respectively, both show consistently poor performance with average scores of 1.21 × 10^1^ and 1.34 × 10^1^. These algorithms fail to win any comparisons against COFOA, further highlighting their relative ineffectiveness. The mSCA, ranked 7th, and WEMFO, ranked 6th, present moderate competition with average scores of 6.32 × 10^0^ and 5.68 × 10^0^ respectively. Both algorithms manage to outperform COFOA in 5 out of 30 runs, indicating a certain level of competitiveness. However, COFOA’s overall superior performance is clear, as evidenced by its consistently lower average score and higher ranking. Other notable algorithms include CLACO and QCSCA, ranked 2nd and 3rd, with average scores of 2.75 × 10^0^ and 3.87 × 10^0^ respectively. Although these algorithms demonstrate relatively strong performance, they still fall short of COFOA, with “+/=/−” metrics of 17/8/5 and 19/4/7, indicating that while they can occasionally match or exceed COFOA’s performance, they do not consistently outperform it.

In addition, the BLPSO and GCHHO algorithms, which rank fourth and fifth, demonstrate relatively competitive performance with mean scores of 3.87 × 10^0^ and 5.32 × 10^0^, respectively. Although these algorithms achieve several victories against COFOA, their higher average scores and lower overall rankings indicate a limited ability to match COFOA’s optimization efficiency. Overall, the experimental results confirm that COFOA consistently outperforms all other compared algorithms on the IEEE CEC 2017 benchmark functions. Its top ranking, combined with the lowest mean performance score, highlights its robustness and effectiveness in addressing complex optimization problems. These results further demonstrate COFOA’s promise as a highly capable framework for practical optimization applications.

#### 4.5.2. Comparative Experiments at CEC 2022 Benchmark Functions

The competing algorithms involved in this experiment include HGWO [24], WEMFO [25], mSCA [26], SCADE [27], CCMWOA [28], QCSCA [29], BWOA [30], CCMSCSA [31], CLACO [35], BLPSO [33], GCHHO [34]. Table 7 provides a comprehensive comparison of COFOA with other state-of-the-art algorithms based on the IEEE CEC 2022 benchmark functions. The analysis includes each algorithm’s ranking, performance relative to COFOA (denoted by wins/draws/losses, +/=/−), and the average performance score (AVG) across multiple runs. In this notation, “+” indicates that COFOA outperforms the compared algorithm, “−” denotes inferior performance, and “=” represents no statistically significant difference. Statistical significance was further assessed using the Wilcoxon signed-rank test [22] and Friedman test [23]. COFOA achieves the top rank with an impressive average score of 2.12 × 10^0^ and is designated as the benchmark algorithm, as indicated by the “~” symbol in the +/=/− column. This result underscores its robust optimization capabilities and consistent ability to obtain optimal solutions across a diverse set of benchmark functions. Among the competing algorithms, QCSCA ranks second with an average score of 2.67 × 10^0^, demonstrating competitive performance with a +/=/− metric of 6/0/6, reflecting instances of both outperformance and underperformance relative to COFOA. Other algorithms, including HGWO, BWOA, and BLPSO, are ranked ninth, eighth, and fifth, with average scores of 7.32 × 10^0^, 7.11 × 10^0^, and 4.76 × 10^0^, respectively. Their performance metrics (10/1/1, 9/2/1, and 8/2/2 +/=/−) show occasional competitiveness against COFOA, but higher average scores indicate less stable performance overall. CLACO and GCHHO, ranked third and fourth, also provide strong competition, with average scores of 3.43 × 10^0^ and 4.49 × 10^0^. Specifically, CLACO’s 4/2/6 +/=/− metric reflects mixed performance, while GCHHO’s 7/2/3 +/=/− demonstrates several instances of competitive advantage. The remaining algorithms, including WEMFO, mSCA, SCADE, CCMWOA, and CCMSCSA, ranked sixth, seventh, twelfth, eleventh, and tenth, respectively, exhibit variable performance relative to COFOA, with successes and failures across different optimization tasks. Overall, these results demonstrate that COFOA consistently outperforms many alternative algorithms on the IEEE CEC 2022 benchmarks, highlighting its robustness, efficiency, and suitability for global optimization problems. These findings further establish COFOA as a leading optimization framework with substantial potential for application in diverse practical domains.

## 5. Application to Oil Reservoir Production

Reservoir production optimization is a critical task in reservoir management, with the principal aim of determining operational strategies that maximize economic returns, quantified by the Net Present Value (NPV). The problem is inherently complex due to the numerous control variables associated with each well over multiple discrete time steps, resulting in a high-dimensional, non-convex, and NP-hard search space. This complexity renders deterministic optimization methods less effective, thereby establishing metaheuristic algorithms as a pragmatic and widely adopted alternative for tackling such challenging optimization landscapes.

To assess the practical efficacy of the proposed COFOA, a series of computational experiments are conducted within a standard industry framework. The widely adopted Eclipse reservoir simulator is employed to model fluid flow dynamics in a synthetic, yet geologically realistic, reservoir model. Within this environment, the COFOA is tasked with optimizing the well control variables, with its performance benchmarked against established metaheuristics including HGWO, mSCA, and SCADE, among others.

The core objective function guiding the optimization is the maximization of NPV, formulated as a discounted cash flow over the production horizon:

(19) $$NPV\left(x,z\right)=\sum_{t=1}^{n}\Delta t\frac{Q_{o,t}\cdot r_{o}-Q_{w,t}\cdot r_{w}-Q_{i,t}\cdot r_{i}}{{\left(1+b\right)}^{p_{t}}}$$

Net Present Value (NPV) is adopted as the objective function to evaluate the economic performance of the reservoir development strategy over the entire production horizon. It is defined as the discounted sum of net cash flows, where $p_{o}$ denotes the oil price, and $q_{o}(t)$ represents the oil production rate at time step t, contributing to the total revenue. The terms $c_{wi}$ and $q_{wi}(t)$ correspond to the unit cost and rate of water injection, respectively, while $c_{wp}$ and $q_{wp}(t)$ denote the cost and amount of produced water, reflecting the operational expenses associated with reservoir management. The parameter r is the annual discount rate, which accounts for the time value of money, ensuring that earlier revenues are weighted more heavily than future cash flows, and τ is a time normalization factor used to maintain consistency in time units. Through this formulation, NPV provides a comprehensive measure that simultaneously captures production benefits, operational costs, and temporal economic effects, making it a suitable and widely accepted objective for optimizing well control strategies in complex reservoir systems.

In this study, the variable x denotes the set of parameters subject to optimization, which, in the current experiment, correspond to the injection and production rates of individual wells. The variable z represents the state parameters of the model, describing the construction of the numerical reservoir system. The total simulation time is indicated by nnn, and $Q_{o,t}$, $Q_{w,t}$, and $Q_{i,t}$ correspond to the oil production rate, water production rate, and water injection rate, respectively, at each time step t. Here, $r_{o}$ represents oil revenue, while $r_{w}$ and $r_{i}$ denote the costs of water treatment and injection. The variable b indicates the average annual interest rate, and $p_{t}$ refers to the elapsed years. During COFOA optimization, each individual in the population represents a candidate solution x, and the fitness function is defined as the net present value (NPV), guiding the population updates in the objective space.

### 5.1. Reservoir Model Description

**Figure 1:**
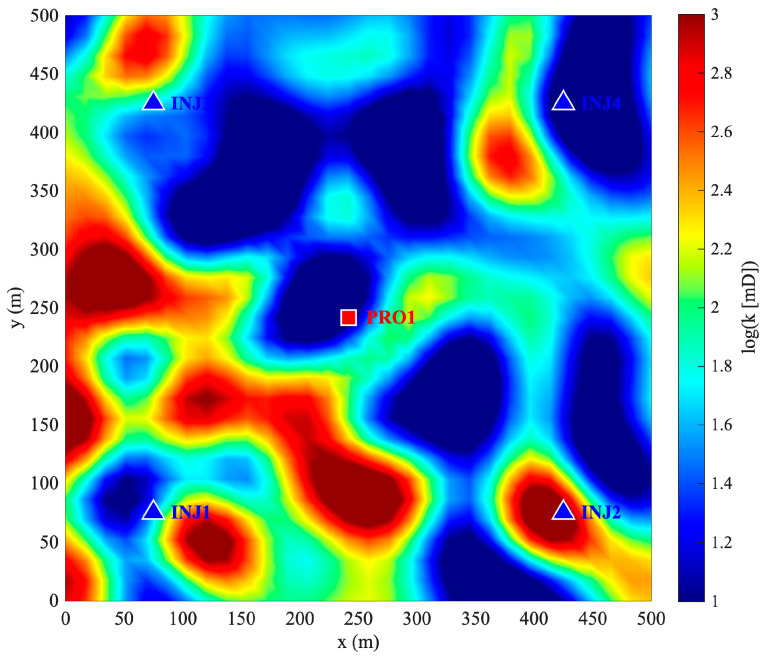
A two-dimensional heterogeneous synthetic reservoir model.

A two-dimensional heterogeneous synthetic reservoir model is developed to represent a complex fluvial channel system. It utilizes a standard five-spot pattern with one central production well (PRO1) and four injection wells (INJ1–INJ4), as illustrated in Figure 1. This model serves to evaluate the proposed optimization algorithm’s efficacy under realistic heterogeneous reservoir conditions.

The spatial domain is discretized into a 30 × 30 Cartesian grid, yielding 900 active cells. Each grid block has uniform thickness and measures roughly 20 m × 20 m. Porosity is assumed constant at 0.2, while permeability is characterized by a stochastically generated field that incorporates high-permeability channels and low-permeability barriers. Figure 1 displays the distribution of log-permeability ln(K), which dictates the predominant fluid flow pathways.

The optimization study spans a production horizon of 1500 days, divided into 10 discrete control steps. Optimization may involve 5 wells, resulting in 50 decision variables depending on the configuration. The objective is to maximize Net Present Value (NPV) by optimizing control settings per well at each step. Economic parameters are assigned reasonable ranges: oil price at 60 USD/STB, water injection and processing costs at 5 USD/STB each, and an annual discount rate of 5%.

### 5.2. Analysis and Discussion of Experimental Results

The performance of the proposed COFOA was comprehensively evaluated against eleven advanced metaheuristic algorithms on the described reservoir model. Each method was executed for five independent runs on the production optimization problem, and the statistical metrics, including the mean, standard deviation (Std), best, and worst Net Present Value (NPV), are summarized for comparison.

Table 8 summarizes the quantitative results obtained by all compared algorithms. Overall, the proposed COFOA achieves the highest mean Net Present Value (NPV) of 9.874 × 10^8^ USD among all methods. In addition, it records the smallest standard deviation (2.152 × 10^7^), indicating superior solution stability and robustness across independent runs. Several competing algorithms, including mSCA, QCSCA, and CCMSCSA, also demonstrate competitive mean NPVs; however, their corresponding standard deviations are notably higher, reflecting increased variability in optimization performance. In contrast, algorithms such as BWOA and SCADE exhibit relatively lower mean NPVs accompanied by larger standard deviations, suggesting weaker consistency and less reliable convergence behavior. The experimental results presented in Table 8 demonstrate the effectiveness of the proposed algorithm in solving the production optimization problem from both economic performance and stability perspectives. Specifically, COFOA achieves the highest mean NPV of $9.874\times 10^{8}$ USD, outperforming all compared algorithms, which indicates its superior ability to identify high-quality well control strategies that maximize long-term economic returns. In addition, COFOA also attains the best maximum NPV of $1.003\times 10^{9}$ USD, further confirming its strong global search capability and potential to reach near-optimal solutions. In contrast, other competitive algorithms such as BLPSO ($9.511\times 10^{8}$) and mSCA ($9.647\times 10^{8}$) exhibit relatively good performance but still fall short of COFOA in both average and best-case results. From the perspective of robustness, COFOA maintains a relatively low standard deviation ($2.152\times 10^{7}$), which is smaller than most algorithms such as WEMFO and QCSCA, indicating more stable convergence behavior and less sensitivity to stochastic factors. Moreover, the worst-case performance of COFOA ($9.652\times 10^{8}$) remains significantly higher than the mean values of many other algorithms, suggesting strong reliability in avoiding poor solutions and mitigating the risk of premature convergence. In comparison, several algorithms such as SCADE and CCMSCSA show lower mean NPVs and larger variances, implying weaker exploration capabilities and a higher tendency to be trapped in local optima, especially under complex heterogeneous reservoir conditions. Overall, these results indicate that COFOA achieves a better balance between exploration and exploitation, enabling it to effectively navigate the high-dimensional and nonlinear search space of the production optimization problem, and ultimately deliver superior economic performance in terms of NPV maximization.

**Figure 2:**
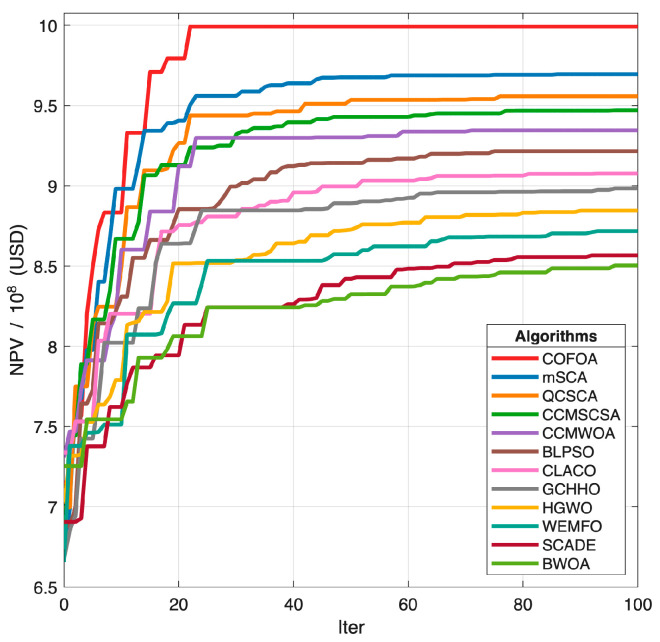
NPV obtained by the algorithms with iteration.

These numerical results are further supported by the convergence curves shown in Figure 2. The COFOA demonstrates a rapid increase in NPV during the early iterations and quickly stabilizes at a higher final level, indicating fast convergence and stable performance. In contrast, the other algorithms generally exhibit slower convergence rates and converge to lower NPV plateaus. Overall, the convergence behavior confirms the effectiveness of COFOA in balancing exploration and exploitation for the production optimization problem.

The proposed COFOA demonstrates superior performance in maximizing NPV for production optimization, achieving both the highest economic return and the most stable convergence among all tested metaheuristics. Its leading efficacy is attributed to a well-balanced exploration-exploitation mechanism, which efficiently navigates the complex search space of well controls. These results confirm the strong practical applicability of COFOA for complex reservoir management problems and establish a solid foundation for its potential deployment in real-field scenarios, as further discussed in the conclusion.

## 6. Conclusions

In this paper, we introduce a novel evolutionary algorithm, COFOA, which is based on the FOA and incorporates a chaotic exploitation mechanism and an orthogonal learning strategy. The chaotic exploitation mechanism enhances COFOA’s global search capabilities, preventing the algorithm from becoming trapped in local optima. Meanwhile, the orthogonal learning strategy improves COFOA’s local search efficiency, ensuring that the algorithm does not overlook the optimal solution.

To evaluate COFOA’s optimization performance, extensive experiments were conducted. Ablation tests performed on the IEEE CEC 2017 benchmark confirmed the effectiveness of both mechanisms within COFOA. By systematically removing each mechanism, we demonstrated that both significantly contribute to enhancing COFOA’s performance. Scalability experiments validated COFOA’s optimization capabilities across multiple dimensions in large-scale, multi-dimensional problems. Comparative experiments with several other optimization algorithms highlighted COFOA’s superior performance. Finally, COFOA was applied to optimize oil and gas production, showcasing its outstanding ability to address real-world problems.

Despite the promising performance of the proposed COFOA, several limitations should be acknowledged. First, the incorporation of the chaotic exploitation mechanism and orthogonal learning strategy inevitably increases the computational burden, particularly for high-dimensional problems, due to additional evaluation and update operations. Second, the algorithm introduces several control parameters, whose settings may influence performance and require careful tuning for different problem scenarios, indicating a certain degree of parameter sensitivity. Third, although COFOA demonstrates strong optimization capability on benchmark functions and application tasks, its generalization ability to more complex real-world problems still requires further validation.

Despite the promising performance of COFOA, several directions merit further investigation. First, to address the increased computational burden in high-dimensional scenarios, future work will focus on developing more efficient strategies, such as dimensionality reduction, surrogate-assisted evaluation, or parallel computation, to improve scalability while maintaining optimization accuracy. Second, considering the sensitivity of algorithm performance to control parameters, adaptive parameter control mechanisms should be introduced, enabling key parameters to be dynamically adjusted during the evolutionary process, thereby enhancing robustness and reducing manual tuning effort. Third, to extend the applicability of COFOA to more complex real-world problems, it is worthwhile to investigate its extension to dynamic and multi-objective optimization frameworks, where time-varying environments and conflicting objectives are involved. Incorporating dynamic multi-objective optimization mechanisms would allow COFOA to better balance convergence and diversity under changing conditions. These improvements are expected to further enhance the efficiency, adaptability, and generalization capability of the proposed algorithm. In future work, several directions can be explored to address these limitations. On the one hand, lightweight or adaptive mechanisms can be developed to reduce computational complexity, such as selectively activating the orthogonal learning strategy or designing surrogate-assisted evaluation methods. On the other hand, parameter control strategies can be introduced to enhance robustness across different problem domains. Furthermore, extending COFOA to handle large-scale, dynamic, and multi-objective optimization problems is also a promising direction. Finally, integrating COFOA with emerging techniques such as neural architecture search or hybrid optimization frameworks may further improve its applicability and performance in practical scenarios.

## Figures and Tables

**Table 1:** IEEE CEC 2017 benchmark function specifications.

Function Equation	Dim	Optimum
$f_{1}\left(x\right)=x_{1}^{2}+10^{6}\sum_{i=2}^{D}x_{i}^{2}$	30	100
$f_{2}\left(x\right)=\sum_{i=1}^{D}\left|x_{i}^{2}\right|$	30	200
$f_{3}\left(x\right)=\sum_{i=1}^{D}x_{i}^{2}+{\left(\sum_{i=1}^{D}0.5x_{i}^{2}\right)}^{2}+{\left(\sum_{i=1}^{D}0.5x_{i}^{2}\right)}^{4}$	30	300
$f_{4}\left(x\right)=\sum_{i=1}^{D-1}\left(100{\left(x_{i}^{2}-x_{i+1}\right)}^{2}+{\left(x_{i}-1\right)}^{2}\right)$	30	400
$f_{5}\left(x\right)=\sum_{i=1}^{D}\left(x_{i}^{2}-10\cos\left(2\pi x_{i}\right)+10\right)$	30	500
$f_{6}\left(x\right)=g\left(x_{1},x_{2}\right)+g\left(x_{2},x_{3}\right)+\cdots +g\left(x_{D-1},x_{D}\right)+g\left(x_{D},x_{1}\right)$ $g\left(x,y\right)=0.5+\frac{\left(\sin^{2}\left(\sqrt{x^{2}+y^{2}}\right)-0.5\right)}{{\left(1+0.001\left(x^{2}+y^{2}\right)\right)}^{2}}$	30	600
$f_{7}\left(x\right)=\min\left({\sum_{i=1}^{D}\left({\overset{̑}{x}}_{i}-\mu_{0}\right)}^{2},\mathrm{dD}+{s\sum_{i=1}^{D}\left({\overset{̑}{x}}_{i}-\mu_{1}\right)}^{2}\right)+10\left(D-\sum_{i=1}^{D}\cos\left(2\pi {\overset{̑}{z}}_{i}\right)\right)$	30	700
$f_{8}\left(x\right)=\sum_{i=1}^{D}\left(z_{i}^{2}-10\cos\left(2\pi z_{i}\right)+10\right)+{f_{13}}^{*}$	30	800
$f_{9}\left(x\right)=\sin^{2}\left(\pi w_{1}\right)+\sum_{i=1}^{D}{\left(w_{i}-1\right)}^{2}\left[1+10\sin^{2}\left(\pi w_{i}+1\right)\right]+{\left(w_{D}-1\right)}^{2}\left[1+\sin^{2}\left(2\pi w_{D}\right)\right]$	30	900
$f_{10}\left(x\right)=418.9829\times D-\sum_{i=1}^{D}g\left(z_{i}\right){,\;z}_{i}=x_{i}+4.209687462275036e+002$	30	1000
$f_{11}\left(x\right)={\sum_{i=1}^{D}\left(10^{6}\right)}^{\frac{i-1}{D-1}}x_{i}^{2}$	3	1100
$f_{12}\left(x\right)=10^{6}x_{1}^{2}+\sum_{i=2}^{D}x_{i}^{2}$	3	1200
$f_{13}\left(x\right)=-20\exp\left(-0.2\sqrt{\frac{1}{D}\sum_{i=1}^{D}x_{i}^{2}}\right)-\exp\left(\frac{1}{D}\sum_{i=1}^{D}\cos\left(2\pi x_{i}\right)\right)+20+e$	3	1300
$f_{14}\left(x\right)=\sum_{i=1}^{D}\left(\sum_{k=0}^{kmax}\left[a^{k}\cos\left(2\pi b^{k}(x+0.5\right))\right]\right)-D\sum_{k=0}^{kmax}\left[a^{k}\cos\left(2\pi b^{k}.0.5\right)\right]$	4	1400
$f_{15}\left(x\right)=\sum_{i=1}^{D}\frac{x_{i}^{2}}{4000}-\prod_{i=1}^{D}\cos\left(\frac{x_{i}}{\sqrt{i}}\right)+1$	4	1500
$f_{16}\left(x\right)=\frac{10}{D^{2}}{\prod_{i=1}^{D}\left(1+i\sum_{j=1}^{32}\frac{\left|2^{j}x_{i}-round\left(2^{j}x_{i}\right)\right|}{2^{j}}\right)}^{\frac{10}{D^{1.2}}}-\frac{10}{D^{2}}$	4	1600
$f_{17}\left(x\right)={\left|\sum_{i=1}^{D}x_{i}^{2}-D\right|}^{1/4}+\left(0.5\sum_{i=1}^{D}x_{i}^{2}+\sum_{i=1}^{D}x_{i}\right)/D+0.5$	5	1700
$f_{18}\left(x\right)={\left|{\left(\sum_{i=1}^{D}x_{i}^{2}\right)}^{2}-{\left(\sum_{i=1}^{D}x_{i}\right)}^{2}\right|}^{1/4}+\left(0.5\sum_{i=1}^{D}x_{i}^{2}+\sum_{i=1}^{D}x_{i}\right)/D+0.5$	5	1800
$f_{19}\left(x\right)=f_{7}\left(f_{4}\left(x_{1},x_{2}\right)\right)+f_{7}\left(f_{4}\left(x_{2},x_{3}\right)\right)+\cdots +f_{7}\left(f_{4}\left(x_{D-1},x_{D}\right)\right)+f_{7}\left(f_{4}\left(x_{D},x_{1}\right)\right)$	5	1900
$f_{20}\left(x\right)={\left[\frac{1}{D-1}\sum_{i=1}^{D-1}\left(\sqrt{s_{i}}\cdot \left(\sin\left(50.0s_{i}^{0.2}\right)+1\right)\right)\right]}^{2},s_{i}=\sqrt{x_{i}^{2}+x_{i+1}^{2}}$	6	2000
$f_{21}\left(x\right)=f_{1}\left(M\left(x-o_{1}\right)\right)+{f_{21}}^{*}$	3	2100
$f_{22}\left(x\right)=f_{2}\left(M\left(x-o_{2}\right)\right)+{f_{22}}^{*}$	3	2200
$f_{23}\left(x\right)=f_{3}\left(M\left(x-o_{3}\right)\right)+{f_{23}}^{*}$	4	2300
$f_{24}\left(x\right)=f_{4}\left(M\left(\frac{2.048\left(x-o_{4}\right)}{100}\right)+1\right)+{f_{24}}^{*}$	4	2400
$f_{25}\left(x\right)=f_{5}\left(M\left(x-o_{5}\right)\right)+{f_{25}}^{*}$	5	2500
$f_{26}\left(x\right)=f_{20}\left(M\left(\frac{2.048\left(x-o_{6}\right)}{100}\right)\right)+{f_{26}}^{*}$	5	2600
$f_{27}\left(x\right)=f_{7}\left(M\left(\frac{600\left(x-o_{7}\right)}{100}\right)\right)+{f_{27}}^{*}$	6	2700
$f_{28}\left(x\right)=f_{8}\left(\frac{5.12\left(x-o_{8}\right)}{100}\right)+{f_{28}}^{*}$	6	2800
$f_{29}\left(x\right)=f_{9}\left(M\left(\frac{5.12\left(x-o_{9}\right)}{100}\right)\right)+{f_{29}}^{*}$	3	2900
$f_{30}\left(x\right)=f_{30}\left(M\left(\frac{1000\left(x-o_{10}\right)}{100}\right)\right)+{f_{30}}^{*}$	3	3000

**Table 2:** IEEE CEC 2022 benchmark function specifications.

Functions	Describe	$f_{i}$
F1	full Rotated Zakharov	300
F2	full Rotated Rosenbrock	400
F3	full Rotated Expanded Schaffer’s f6	600
F4	full Rotated Non-Continuous Restrain	800
F5	full Rotated Levy	900
F6	Hybrid	1800
F7	Hybrid	2000
F8	Hybrid	2200
F9	Composition	2300
F10	Composition	2400
F11	Composition	2600
F12	Composition	2700

**Table 3:** Ablation analysis.

Algorithm	Rank	+/−/=	AVG
**COFOA**	**1**	**~**	**1.8**
CFOA	3	8/3/19	2.8
OFOA	2	18/5/7	2.5
FOA	4	15/3/12	2.9

**Table 4:** Scalability tests in three dimensions.

	Dim	30		50		100	
	Metric	COFOA	FOA	COFOA	FOA	COFOA	FOA
**F1**	AVG	5.15 × 10^3^	1.06 × 10^6^	3.69 × 10^3^	2.21 × 10^6^	1.03 × 10^4^	4.91 × 10^6^
**F2**	AVG	6.79 × 10^9^	4.56 × 10^13^	1.56 × 10^32^	1.43 × 10^31^	3.41 × 10^66^	4.81 × 10^96^
**F3**	AVG	5.32 × 10^3^	1.61 × 10^3^	4.93 × 10^4^	1.12 × 10^4^	2.42 × 10^5^	7.40 × 10^4^
**F4**	AVG	4.80 × 10^2^	4.99 × 10^2^	5.17 × 10^2^	5.52 × 10^2^	6.27 × 10^2^	6.73 × 10^2^
**F5**	AVG	5.43 × 10^2^	5.37 × 10^2^	6.21 × 10^2^	5.86 × 10^2^	1.22 × 10^3^	1.13 × 10^3^
**F6**	AVG	6.00 × 10^2^	6.00 × 10^2^	6.00 × 10^2^	6.00 × 10^2^	6.00 × 10^2^	6.00 × 10^2^
**F7**	AVG	8.04 × 10^2^	7.74 × 10^2^	9.75 × 10^2^	8.94 × 10^2^	1.53 × 10^3^	1.52 × 10^3^
**F8**	AVG	8.46 × 10^2^	8.39 × 10^2^	9.27 × 10^2^	9.00 × 10^2^	1.49 × 10^3^	1.37 × 10^3^
**F9**	AVG	9.03 × 10^2^	9.23 × 10^2^	9.07 × 10^2^	9.64 × 10^2^	9.08 × 10^2^	1.29 × 10^3^
**F10**	AVG	4.13 × 10^3^	2.98 × 10^3^	9.18 × 10^3^	5.94 × 10^3^	2.45 × 10^4^	2.38 × 10^4^
**F11**	AVG	1.13 × 10^3^	1.70 × 10^3^	1.15 × 10^3^	1.30 × 10^3^	1.66 × 10^3^	2.10 × 10^3^
**F12**	AVG	2.27 × 10^5^	2.54 × 10^6^	1.46 × 10^6^	1.09 × 10^7^	2.89 × 10^6^	2.56 × 10^7^
**F13**	AVG	1.58 × 10^4^	1.03 × 10^6^	6.95 × 10^3^	2.41 × 10^6^	4.48 × 10^3^	4.22 × 10^5^
**F14**	AVG	6.44 × 10^4^	8.78 × 10^5^	9.37 × 10^4^	2.21 × 10^6^	3.26 × 10^5^	3.58 × 10^6^
**F15**	AVG	1.13 × 10^4^	5.28 × 10^4^	9.53 × 10^3^	2.66 × 10^5^	5.90 × 10^3^	2.43 × 10^5^
**F16**	AVG	2.34 × 10^3^	2.39 × 10^3^	2.87 × 10^3^	2.64 × 10^3^	5.86 × 10^3^	4.88 × 10^3^
**F17**	AVG	2.07 × 10^3^	2.04 × 10^3^	2.57 × 10^3^	2.49 × 10^3^	5.06 × 10^3^	3.93 × 10^3^
**F18**	AVG	2.20 × 10^5^	2.11 × 10^6^	1.30 × 10^6^	3.48 × 10^6^	2.58 × 10^6^	3.16 × 10^6^
**F19**	AVG	1.20 × 10^4^	6.30 × 10^4^	2.11 × 10^4^	8.83 × 10^4^	4.29 × 10^3^	3.11 × 10^5^
**F20**	AVG	2.36 × 10^3^	2.40 × 10^3^	2.71 × 10^3^	2.66 × 10^3^	1.03 × 10^4^	4.91 × 10^6^
**F21**	AVG	2.34 × 10^3^	2.34 × 10^3^	2.43 × 10^3^	2.39 × 10^3^	4.96 × 10^3^	5.90 × 10^3^
**F22**	AVG	2.30 × 10^3^	3.10 × 10^3^	9.94 × 10^3^	7.92 × 10^3^	3.00 × 10^3^	2.76 × 10^3^
**F23**	AVG	2.71 × 10^3^	2.72 × 10^3^	2.83 × 10^3^	2.84 × 10^3^	2.89 × 10^4^	2.50 × 10^4^
**F24**	AVG	2.88 × 10^3^	2.88 × 10^3^	3.06 × 10^3^	3.07 × 10^3^	3.03 × 10^3^	3.02 × 10^3^
**F25**	AVG	2.89 × 10^3^	2.90 × 10^3^	3.01 × 10^3^	3.05 × 10^3^	3.53 × 10^3^	3.59 × 10^3^
**F26**	AVG	3.94 × 10^3^	4.00 × 10^3^	4.75 × 10^3^	4.45 × 10^3^	3.28 × 10^3^	3.34 × 10^3^
**F27**	AVG	3.23 × 10^3^	3.24 × 10^3^	3.41 × 10^3^	3.47 × 10^3^	1.06 × 10^4^	7.84 × 10^3^
**F28**	AVG	3.16 × 10^3^	3.23 × 10^3^	3.29 × 10^3^	3.31 × 10^3^	3.49 × 10^3^	3.63 × 10^3^
**F29**	AVG	3.58 × 10^3^	3.72 × 10^3^	3.84 × 10^3^	3.78 × 10^3^	3.38 × 10^3^	3.42 × 10^3^
**F30**	AVG	1.07 × 10^4^	1.90 × 10^5^	9.96 × 10^5^	1.19 × 10^6^	5.32 × 10^3^	4.38 × 10^3^
**+/−/=**	~	~	17/6/7	~	14/8/8	~	15/10/5

**Table 5:** Parameter sensitivity analysis.

F1										
Popsize	10		30		60		100		200	
	Avg	Std	Avg	Std	Avg	Std	Avg	Std	Avg	Std
COFOA	8.0 × 10^2^	3.0 × 10^1^	6.3 × 10^2^	4.0 × 10^1^	5.9 × 10^2^	2.3 × 10^1^	5.8 × 10^2^	2.6 × 10^1^	5.7 × 10^2^	1.7 × 10^1^
FOA	9.0 × 10^2^	8.2 × 10^1^	6.7 × 10^2^	4.2 × 10^1^	6.3 × 10^2^	4.6 × 10^1^	6.4 × 10^2^	4.2 × 10^1^	6.9 × 10^2^	5.3 × 10^1^
F2										
Popsize	10		30		60		100		200	
	Avg	Std	Avg	Std	Avg	Std	Avg	Std	Avg	Std
COFOA	1.0 × 10^4^	2.9 × 10^4^	7.3 × 10^3^	6.2 × 10^3^	1.6 × 10^4^	1.3 × 10^4^	1.1 × 10^4^	8.8 × 10^3^	1.2 × 10^4^	1.3 × 10^4^
FOA	8.7 × 10^4^	5.5 × 10^4^	7.3 × 10^4^	3.9 × 10^4^	1.1 × 10^5^	7.2 × 10^4^	9.8 × 10^4^	5.8 × 10^4^	1.2 × 10^8^	3.7 × 10^8^
F3										
Popsize	10		30		60		100		200	
	Avg	Std	Avg	Std	Avg	Std	Avg	Std	Avg	Std
COFOA	1.8 × 10^5^	2.7 × 10^5^	2.3 × 10^4^	9.0 × 10^3^	1.6 × 10^4^	6.2 × 10^3^	1.7 × 10^4^	1.0 × 10^4^	1.6 × 10^4^	1.4 × 10^4^
FOA	1.3 × 10^7^	1.1 × 10^7^	7.9 × 10^6^	4.5 × 10^6^	6.2 × 10^6^	6.6 × 10^6^	1.1 × 10^7^	1.1 × 10^7^	5.5 × 10^7^	1.4 × 10^8^

**Table 6:** Experiments comparing COFOA with alternative competing algorithms on the IEEE CEC 2017 benchmark functions.

Algorithm	Rank	+/=/−	AVG
**COFOA**	**1**	**~**	**1.84 × 10^0^**
HGWO	9	30/0/0	9.12 × 10^0^
mSCA	7	26/0/4	6.32 × 10^0^
SCADE	11	30/0/0	1.21 × 10^1^
CCMWOA	12	30/0/0	1.34 × 10^1^
WEMFO	6	25/0/5	5.68 × 10^0^
CCMSCSA	8	29/0/1	8.98 × 10^0^
SSNMRA	2	10/13/7	2.18 × 10^0^
QCSCA	3	18/4/8	3.87 × 10^0^
BWOA	10	30/0/0	9.32 × 10^0^
BLPSO	4	22/1/7	3.87 × 10^0^
GCHHO	5	21/2/7	5.32 × 10^0^

**Table 7:** Experiments comparing COFOA with alternative competing algorithms at IEEE CEC 2022 benchmark functions.

Algorithm	Rank	+/=/−	AVG
**COFOA**	**1**	**~**	**2.12 × 10^0^**
WEMFO	6	9/2/1	5.11 × 10^0^
mSCA	7	11/0/1	5.89 × 10^0^
SCADE	12	10/2/0	8.36 × 10^0^
CCMWOA	11	9/3/0	8.24 × 10^0^
QCSCA	2	7/0/5	2.67 × 10^0^
HGWO	9	10/1/1	7.32 × 10^0^
BWOA	8	9/2/1	7.11 × 10^0^
BLPSO	5	6/2/1	4.76 × 10^0^
CCMSCSA	10	11/1/0	8.56 × 10^0^
CLACO	3	4/2/6	3.43 × 10^0^
GCHHO	4	8/2/2	4.49 × 10^0^

**Table 8:** Experimental Results of All Algorithms on the Production Optimization Problem.

Algorithm	Mean (USD)	Std	Best	Worst
COFOA	9.874 × 10^8^	2.152 × 10^7^	1.003 × 10^9^	9.652 × 10^8^
WEMFO	8.812 × 10^8^	5.113 × 10^7^	9.208 × 10^8^	8.253 × 10^8^
mSCA	9.647 × 10^8^	2.531 × 10^7^	9.872 × 10^8^	9.381 × 10^8^
SCADE	8.563 × 10^8^	5.824 × 10^7^	8.994 × 10^8^	7.905 × 10^8^
CCMWOA	9.305 × 10^8^	3.540 × 10^7^	9.612 × 10^8^	8.942 × 10^8^
QCSCA	8.691 × 10^8^	5.467 × 10^7^	9.102 × 10^8^	8.092 × 10^8^
HGWO	9.423 × 10^8^	3.286 × 10^7^	9.698 × 10^8^	9.105 × 10^8^
BWOA	9.038 × 10^8^	4.502 × 10^7^	9.394 × 10^8^	8.545 × 10^8^
BLPSO	9.511 × 10^8^	3.017 × 10^7^	9.745 × 10^8^	9.214 × 10^8^
CCMSCSA	8.495 × 10^8^	6.021 × 10^7^	8.936 × 10^8^	7.823 × 10^8^
CLACO	9.176 × 10^8^	4.126 × 10^7^	9.503 × 10^8^	8.711 × 10^8^
GCHHO	8.945 × 10^8^	4.871 × 10^7^	9.321 × 10^8^	8.410 × 10^8^

## Data Availability

Data is available upon request.

## References

[1] Wiggins M.L., Startzman R.A. (1990). An Approach to Reservoir Management. SPE Annual Technical Conference and Exhibition?.

[2] Lake L.W., Johns R.T., Rossen W.R., Pope G.A. (2014). Fundamentals of Enhanced Oil Recovery.

[3] Rao S.S. (2019). Engineering Optimization: Theory and Practice.

[4] Liu X., Reynolds A.C. (2014). Gradient-Based Multiobjective Optimization with Applications to Waterflooding Optimization. ECMOR XIV-14th European Conference on the Mathematics of Oil Recovery.

[5] Al-Aghbari M., Al-Wadhahi M., Gujarathi A.M. (2022). Multi-Objective Optimization of Brugge Field for Short-Term and Long-Term Waterflood Management. Arab. J. Sci. Eng..

[6] Puspita T. (2025). Bayesian Hyperparameter Optimization Analysis for Sustainable Innovation Performance Prediction Model. Eig. Math. J..

[7] Mirjalili S. (2015). Moth-Flame Optimization Algorithm: A Novel Nature-Inspired Heuristic Paradigm. Knowl.-Based Syst..

[8] Kennedy J., Eberhart R. (1995). Particle Swarm Optimization. IEEE International Conference on Neural Networks—Conference Proceedings.

[9] Yang X.S. (2010). A New Metaheuristic Bat-Inspired Algorithm. Studies in Computational Intelligence.

[10] Mirjalili S. (2016). Sca: A Sine Cosine Algorithm for Solving Optimization Problems. Knowl.-Based Syst..

[11] Pan W.T. (2012). A New Fruit Fly Optimization Algorithm: Taking the Financial Distress Model as an Example. Knowl.-Based Syst..

[12] Wolpert D.H., Macready W.G. (1997). No Free Lunch Theorems for Optimization. IEEE Trans. Evol. Comput..

[13] Mehmood K., Chaudhary N.I., Khan Z.A., Cheema K.M., Raja M.A.Z. (2023). Variants of Chaotic Grey Wolf Heuristic for Robust Identification of Control Autoregressive Model. Biomimetics.

[14] Cao Y., Xu Y., Du X., Zhong R., Yu J., Munetomo M. (2025). Tri-Subpopulation Sigmoid-Enhanced Sine-Cosine Algorithm and Its Application to Gene Function Prediction Problem. J. Supercomput..

[15] Cao Y., Du X., Yu J., Zhong R., Munetomo M. (2025). Dynamic Fitness-Distance Balance Competitive Swarm Optimizer: Performance Investigation, Engineering Simulation, and Application in Superconductor Critical Temperature Prediction. Clust. Comput..

[16] Wang Z.-Z., Zhang K., Chen G.-D., Zhang J.-D., Wang W.-D., Wang H.-C., Zhang L.-M., Yan X., Yao J. (2023). Evolutionary-Assisted Reinforcement Learning for Reservoir Real-Time Production Optimization under Uncertainty. Pet. Sci..

[17] Du S.-Y., Zhao X.-G., Xie C.-Y., Zhu J.-W., Wang J.-L., Yang J.-S., Song H.-Q. (2023). Data-Driven Production Optimization Using Particle Swarm Algorithm Based on the Ensemble-Learning Proxy Model. Pet. Sci..

[18] Ng C.S.W., Ghahfarokhi A.J., Amar M.N. (2023). Production Optimization under Waterflooding with Long Short-Term Memory and Metaheuristic Algorithm. Petroleum.

[19] Zhou Q., Dai R., Zhou G., Ma S., Luo S. (2024). An Enhanced Tree-Seed Algorithm for Function Optimization and Production Optimization. Biomimetics.

[20] Chen H., Li S., Heidari A.A., Wang P., Li J., Yang Y., Wang M., Huang C. (2020). Efficient Multi-Population Outpost Fruit Fly-Driven Optimizers: Framework and Advances in Support Vector Machines. Expert Syst. Appl..

[21] Abed-Alguni B.H., Alawad N.A., Barhoush M., Hammad R. (2021). Exploratory Cuckoo Search for Solving Single-Objective Optimization Problems. Soft Comput..

[22] Demsar J. (2006). Statistical Comparisons of Classifiers over Multiple Data Sets. J. Mach. Learn. Res..

[23] García S., Fernández A., Luengo J., Herrera F. (2010). Advanced Nonparametric Tests for Multiple Comparisons in the Design of Experiments in Computational Intelligence and Data Mining: Experimental Analysis of Power. Inf. Sci..

[24] Deng S., Wang X., Zhu Y., Lv F., Wang J. (2019). Hybrid Grey Wolf Optimization Algorithm–Based Support Vector Machine for Groutability Prediction of Fractured Rock Mass. J. Comput. Civ. Eng..

[25] Shan W., Qiao Z., Heidari A.A., Chen H., Turabieh H., Teng Y. (2021). Double Adaptive Weights for Stabilization of Moth Flame Optimizer: Balance Analysis, Engineering Cases, and Medical Diagnosis. Knowl.-Based Syst..

[26] Gupta S., Deep K. (2019). A Hybrid Self-Adaptive Sine Cosine Algorithm with Opposition Based Learning. Expert Syst. Appl..

[27] Alambeigi F., Aghajani Pedram S., Speyer J.L., Rosen J., Iordachita I., Taylor R.H., Armand M. (2020). Scade: Simultaneous Sensor Calibration and Deformation Estimation of Fbg-Equipped Unmodeled Continuum Manipulators. IEEE Trans. Robot..

[28] Luo J., Chen H., Heidari A.A., Xu Y., Zhang Q., Li C. (2019). Multi-Strategy Boosted Mutative Whale-Inspired Optimization Approaches. Appl. Math. Model..

[29] Hu H., Shan W., Tang Y., Heidari A.A., Chen H., Liu H., Wang M., Escorcia-Gutierrez J., Mansour R.F., Chen J. (2022). Horizontal and Vertical Crossover of Sine Cosine Algorithm with Quick Moves for Optimization and Feature Selection. J. Comput. Des. Eng..

[30] Srikanth Reddy K., Panwar L., Panigrahi B.K., Kumar R. (2019). Binary Whale Optimization Algorithm: A New Metaheuristic Approach for Profit-Based Unit Commitment Problems in Competitive Electricity Markets. Eng. Optim..

[31] Shan W., Hu H., Cai Z., Chen H., Liu H., Wang M., Teng Y. (2022). Multi-Strategies Boosted Mutative Crow Search Algorithm for Global Tasks: Cases of Continuous and Discrete Optimization. J. Bionic Eng..

[32] Singh S., Singh H., Mittal N., Punj G.K., Kumar L., Fante K.A. (2025). A Hybrid Swarm Intelligent Optimization Algorithm for Antenna Design Problems. Sci. Rep..

[33] Chen X., Li K., Xu B., Yang Z. (2020). Biogeography-Based Learning Particle Swarm Optimization for Combined Heat and Power Economic Dispatch Problem. Knowl.-Based Syst..

[34] Song S., Wang P., Heidari A.A., Wang M., Zhao X., Chen H., He W., Xu S. (2021). Dimension Decided Harris Hawks Optimization with Gaussian Mutation: Balance Analysis and Diversity Patterns. Knowl.-Based Syst..

[35] Liu L., Zhao D., Yu F., Heidari A.A., Li C., Ouyang J., Chen H., Mafarja M., Turabieh H., Pan J. (2021). Ant Colony Optimization with Cauchy and Greedy Levy Mutations for Multilevel Covid 19 X-Ray Image Segmentation. Comput. Biol. Med..

